# Supplementary material for: Promoting the implementation of clinical decision support systems in primary care: A qualitative exploration of implementing a Fractional exhaled Nitric Oxide (FeNO)-guided decision support system in asthma consultations
Source: PLoS One. 2025 Feb 13;20(2):e0317613. doi: 10.1371/journal.pone.0317613 (PMC11824951; doi:10.1371/journal.pone.0317613)
Supplement: S3 File — (DOCX) [file pone.0317613.s003.docx]

**
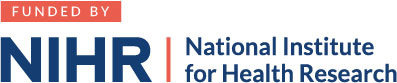

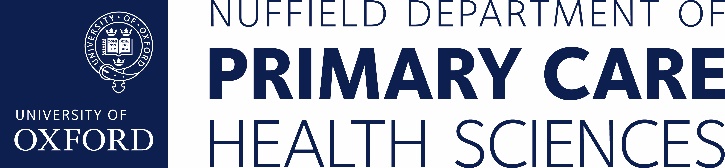
**

**Additional file 3. Topic guide for healthcare professional interviews during the feasibility study**

1. Welcome and thanks to participant for agreeing to take part.
2. Introduce self.
3. This interview is for the DEFINE study. The aims are described in the participant information sheet. Differences between professionals may arise from different perceptions of settings and organisations, from different priorities, and from different beliefs about healthcare provision. These differences are important to us and we value your unique perspective.
4. If at any time during the interview you do not wish to answer a question and that is okay.
5. I would like to audio record our conversation. The recording will be transcribed, but your data will be pseudonymised. Your name and any names you mention, and any places you mention will be taken out, so that if someone read your interview transcript, they would not know who you are or where you work.
6. Your interview will remain confidential.
7. If, at any stage, you wish to stop the audio recording, please let me know.
8. Do you have any questions?

## Topics to be explored

Below is a list of topics to be discussed. The topic guide will remain flexible with respect to what is of importance to participants. However, the key topic of health care professionals’ views and experiences of using fractional exhaled nitric oxide (FeNO) during asthma review consultations in primary care will remain the same. Some topics may also be used to interview other general practice staff if they have a role in the implementation of FeNO in their general practice.

1. Completing the FeNO web-based training.
2. Measuring FeNO during the consultation.
3. Using the FeNO web tool to guide clinical decisions about managing patients asthma.
4. Barriers and facilitators to the implementation of the FeNO-guided intervention in primary care.

**Example questions**:

1. What was your role during the study?
   1. Could you tell me more about the activities you took part in? Overall how did the study go?
   2. Who were the other HCPs involved in the study? What is their job role?
   3. How did you work with other HCPs on the study?
2. Before you took part in this study, what were your views and experience of using FeNO to guide clinical decisions in patients with asthma?
   1. Have you ever used a FeNO test before?
3. What did you think of the FeNO training? This was the online module you completed at the start of the study,
   1. How helpful was the training to understand how to use the FeNO web tool? How was the length of the training? Was there anything missing?
4. Were you involved in identifying patients to invite to the study?
   1. If so, how did you decide who to invite?
5. How did you find the process of measuring FeNO during the consultation?
   1. Have you experienced any barriers? How did you overcome them?
   2. How did your patients find doing the FeNO test?
   3. Have you encountered any barriers in storing or accessing the FeNO analyser?
   4. Have you encountered any technical issues with the machine?
   5. Have you used the FeNO handout which shows you how to take a reading?
   6. If yes: How did you use it? Have you used the handout with your patients? What did they think of the handout?
6. What was your experience of explaining FeNO to patients?
   1. Have you experienced any barriers? How did you overcome them?
   2. What were the most commons questions from patients?
   3. What did you think about sending a leaflet about FeNO testing to patients before the asthma review?
   4. Do you think your patients had read it?
7. What did your patients think of you using their FeNO test result to guide decisions about managing their asthma?
8. How did knowing your patient’s FeNO test result help you?
   1. Could you tell me about a time when it helped you?
   2. Could you tell me about a time when it did not help you?
9. How did you find using the FeNO web tool during asthma reviews? This was the online tool where you entered patient’s FeNO reading, asthma exacerbations and asthma control test to receive recommendations.
   1. How did the FeNO web tool influence your clinical decision-making?
   2. Have you experienced any barriers? How did you overcome them?
   3. How did you find the recommendations of the web tool? Could you tell me of an example of when you did not follow them and why?
   4. Is there anything you would add or change to the web tool?
   5. Have you encountered any technical issues using the web tool during the asthma review?
   6. Do you think the web tool changed the way you would normally conduct a review? Can you tell me why?
   7. What did your patients think of you using the FeNO web tool to guide decisions about managing their asthma?
10. What about any changes to patients’ medication following the review?

Can you talk me through how that worked at your Practice?

1. Having taken part in this study, what are your views of implementing FeNO during asthma review consultations in primary care?
   1. Have you experienced any barriers in implementing FeNO during your asthma reviews? What did you do to overcome them?
2. Do you have anything else you’d like to mention about the study?

Thank you for your time.

Implementation questions at the end:

- Are you a prescriber?
- Did you measure the FeNO test and use the web tool in one review or did you have follow up consultations? Did you have face to face or telephone discussions about the web tool recommendations?
- Did you send patients the patient leaflet? When?
