# Supplementary material for: Promoting the implementation of clinical decision support systems in primary care: A qualitative exploration of implementing a Fractional exhaled Nitric Oxide (FeNO)-guided decision support system in asthma consultations
Source: PLoS One. 2025 Feb 13;20(2):e0317613. doi: 10.1371/journal.pone.0317613 (PMC11824951; doi:10.1371/journal.pone.0317613)
Supplement: S4 File — (DOCX) [file pone.0317613.s004.docx]

Additional file 4. Implementation strategies identified from the analysis defined according to the ERIC taxonomy, CFIR domain being targeted, determinant being addressed, and details about who performs the action, when, how often, and expected implementation outcome being targeted.

| **Specific actions** (bracketed text shows where this strategy was described in the present study)  (Proctor) | **Support system actor** (Leeman) | **Delivery system actor** (Leeman) | **Temporality** (EPIS) (Proctor) | **Frequency** (Proctor) | **Expected implementation outcomes addressed** (Proctor) | **ERIC strategy** (Powell) | **CFIR Implementation domain**  **(and construct)** (Damschroder) | **Determinant being addressed**  (defined as modifiable factors that prevent or enable implementation, Leeman) |
| --- | --- | --- | --- | --- | --- | --- | --- | --- |
| Enable clinicians to override decision support system recommendations if they perceive them to be inappropriate. | Research team | Healthcare practitioner using the intervention | Preparation during study development | Once at study outset | Acceptability | Promote adaptability | Intervention (Adaptability) | *Perceived lack of autonomy and control* |
| Rather than providing only the recommended action for clinicians, include a rationale for how this recommendation was generated and show how it is consistent with evidence and guidelines. | Research team | Healthcare practitioner using the intervention | Implementation | Each time a recommendation is received | Appropriateness  Fidelity | Tailor strategies | Intervention (Evidence strength and quality)  Outer setting (External policies and incentives) | *Lack of confidence about effectiveness or appropriateness of decision support system recommendation to achieve patient outcomes* |
| During set-up, training, and within the decision support system recommendations themselves; demonstrate how use of the decision support system enhances patient outcomes, and facilitate discussion with patient about the recommendations during the consultation. | Research team | Healthcare practitioner using the intervention | Implementation | Before and during implementation  Each time a recommendation is received | Fidelity  Acceptability  Appropriateness | Audit and provide feedback  Inform local opinion leaders  Tailor strategies | Intervention (Relative advantage) | Perceived benefit of using the decision support system for the patient |

**Determinants which prevent implementation are shown in italics.* Determinants which enable implementation are shown as normal text.
