# Supplementary material for: Promoting the implementation of clinical decision support systems in primary care: A qualitative exploration of implementing a Fractional exhaled Nitric Oxide (FeNO)-guided decision support system in asthma consultations
Source: PLoS One. 2025 Feb 13;20(2):e0317613. doi: 10.1371/journal.pone.0317613 (PMC11824951; doi:10.1371/journal.pone.0317613)
Supplement: Attachment — Submitted filename: Response to Reviewers_04.10.24.docx [file pone.0317613.s006.docx]

04/10/24

Dear Reviewers,

Thank you very much for reviewing this paper, we really appreciate your additional suggestions for improvements and have implemented them below. The page and line numbers refer to the location of the change in the tracked copy.

Many thanks

Kate Morton

Corresponding author

| **Reviewer comment** | **Page number, section** | **Response** |
| --- | --- | --- |
| Were these rule based recommendations e.g. based on a decision tree, or generated via other means e.g. Generative AI? | Table 1, page 8, methods | The recommendations were based on a decision tree. This is referred to on page 7:  “The CDSS recommendations were based on an algorithm developed by leading clinicians with expertise in FeNO-guided asthma management (KW, MT and others) via consensus meetings”.  I’ve also made this clearer in Table 1 by renaming the column that was called ‘recommendations’ as ‘recommendations developed by stakeholders as a decision tree’ (page 8) |
| What happens to the CDSS if there's no FeNO results available? | Page 9, methods | Clarified that ‘If no FeNO test result was available, the CDSS could not be used as it required a FeNO result to be inputted in order to generate a recommendation.’ (page 9) |
| Why 4-5 interviews the cut-off for one cycle? | Page 9-10, methods | This batched approach is in line with the person-based approach  to developing digital behavioural interventions. It allows the study team to adapt and optimise interventions and explore the acceptability of such optimisations. A citation has been added to clarify this (page 9-10) |
| How many Think Aloud cycles were done and what was the change across the cycles? | Page 12, methods | There were 4 cycles of thinkaloud interviews, and this has been added to the paper (page 12).  Changes made to the CDSS between batches of thinkaloud interviews are reported elsewhere, and this has been clarified (page 12) |
| Clarify this breakdown  Does this mean 8 clinicians did not had a direct interaction with the CDSS screens but only the online training module? | Page 12, results | This has now been clarified:  “eight clinicians viewed just the online training module with patient scenarios and CDSS recommendations, six only inputted dummy data to the CDSS to view recommendations, and two did both”. (page 12) |
| Suggest a breakdown across the 2 parts - Think Aloud and Process evaluation. What were their experiences in managing asthma, gender distribution, practice size, volume of asthma patients seen each month? | Page 12, results | We have now broken this down between the thinkaloud and feasibility study for the data we have, but unfortunately we did not collect data regarding experience in managing asthma, gender, practice size, or volume of asthma patients seen each month. (page 12) |
| No quotes from GPs seem to have been included, please ensure representation from all the groups is present and negative/dissenting opinions were also included | Page 12, results | Thank you for highlighting this. We have now included some more quotes from GPs to ensure their views are represented and discussed (pages 12-18) |
| How does these results fit within the wider CDSS implementation literature? Some recent relevant papers:  https://pubmed.ncbi.nlm.nih.gov/33965933/  https://pubmed.ncbi.nlm.nih.gov/35673136/  https://doi.org/10.3399/BJGP.2022.0608  https://pubmed.ncbi.nlm.nih.gov/36809791/  As well as when considering common models of technology adoption e.g. UTAUT and TAM. | Page 17, discussion | Thank you for the additional references, which have been read and added in to enhance the discussion and links with current literature (pages 18-22) |
